# Unveiling the synergistic potency of chlorhexidine and azithromycin in combined action

**DOI:** 10.1007/s00210-024-03010-0

**Published:** 2024-02-20

**Authors:** Gizem Samgane, Sevinç Karaçam, Sinem Tunçer Çağlayan

**Affiliations:** 1 Department of Biotechnology, Bilecik Şeyh Edebali University, Bilecik, 11100 Turkey; 2 Central Research and Application Laboratory, Bilecik Şeyh Edebali University, Bilecik, 11100 Turkey; 3 Department of Medical Services and Techniques, Vocational School of Health Services, Bilecik Şeyh Edebali University, Pelitözü Mah. Fatih Sultan Mehmet Bulvarı No:27, Bilecik, 11100 Turkey

**Keywords:** Chlorhexidine, Azithromycin, Growth inhibition, Flagellin, TLR5, NF-κB

## Abstract

The growing challenge of antibiotic resistance necessitates novel approaches for combating bacterial infections. This study explores the distinctive synergy between chlorhexidine, an antiseptic and disinfectant agent, and azithromycin, a macrolide antibiotic, in their impact on bacterial growth and virulence factors using *Escherichia coli* strain Crooks (ATCC 8739) as a model. Our findings reveal that the chlorhexidine and azithromycin combination demonstrates enhanced anti-bacterial effects compared to individual treatments. Intriguingly, the combination induced oxidative stress, decreased flagellin expression, impaired bacterial motility, and enhanced bacterial autoaggregation. Notably, the combined treatment also demonstrated a substantial reduction in bacterial adherence to colon epithelial cells and downregulated NF-κB in the epithelial cells. In conclusion, these results shed light on the potential of the chlorhexidine and azithromycin synergy as a compelling strategy to address the rising challenge of antibiotic resistance and may pave the way for innovative therapeutic interventions in tackling bacterial infections.

**Supplementary Information:**

The online version contains supplementary material available at 10.1007/s00210-024-03010-0.

## Introduction

Antimicrobial resistance (AMR) is a significant problem leading to increased mortality, reduced quality and performance of healthcare facilities, and substantial economic damage (Zhu et al. 2022; Irfan et al. 2022). Despite intense efforts, the required chemical, biological, and pharmacological characteristics for effective antibiotics have hindered the development of new antibiotic classes for years. Moreover, the discovery of new antibiotics has been unable to keep pace with the speed of AMR emergence, while unnecessary global antibiotic use selectively enriches AMR pathogens, further increasing the risk of AMR (Tyers and Wright 2019; Zhu et al. 2022). Given the growing AMR crisis, established strategies in drug discovery and development need to be critically examined to address the gap between the potential and speed of discovering new antibiotics and the need to combat AMR. In this point, the combined application of antimicrobial agents, targeting multiple inhibitory mechanisms and reducing the emergence of spontaneous resistance, can enhance effectiveness and suppress antibacterial resistance compared to the individual effects of agents by creating a synergistic effect. Synergistic interactions have potential advantages for antibacterial efficacy, including bypassing resistance mechanisms and reducing toxicity in the host (Ejim et al. 2011; Tyers and Wright 2019; Zhu et al. 2021).

In this manuscript, the synergistic effect of chlorhexidine (CHX), an antiseptic and disinfectant, with the antibiotic azithromycin (AZM) was examined from various aspects related to pathogenicity. AZM (C_38_H_72_N_2_O_12_) is a second-generation, broad-spectrum, semi-synthetic macrolide antibiotic that has been used since the 1980s to treat respiratory, urogenital, dermal, and many other bacterial infections. Additionally, it acts as an immunomodulator in chronic inflammatory disorders (Parnham et al. 2014). Through reversible interaction with 23S rRNA, macrolides inhibit bacterial protein translation by blocking the peptide exit channel of the 50S ribosomal subunit and thus, interfere with the progression of the growing chain, leading to premature dissociation of incomplete peptide chains. This phenomenon, known as “peptidyl-tRNA drop-off”, depletes the intracellular pools of aminoacyl-tRNA available for protein synthesis (Leroy et al. 2021). While macrolides have been traditionally regarded as tunnel blockers to clog up the ribosomal tunnel and thereby block general protein synthesis, emerging evidence suggests that they selectively inhibit the translation of cellular proteins and their effects are critically dependent on the nascent protein sequence. Therefore, macrolides function as translational modulators rather than global inhibitors of protein synthesis (Vázquez-Laslop and Mankin 2018). Similar to other antibiotics, suboptimal use of AZM (inappropriate dose and/or treatment duration) is considered a significant factor contributing to the development of resistant bacteria (Hooda et al. 2020; Heidary et al. 2022).

While extensive research and publications have been conducted for many years to determine the combined effects of various antibiotic-antibiotic interactions, the effects of using antibiotics together with biocides or antiseptics, which are used as antimicrobials, have not been systematically investigated. In the literature, this is highlighted as a gap, emphasizing the need for studies on syncretic combinations (Ejim et al. 2011; Tyers and Wright 2019; Pietsch et al. 2021; Zhu et al. 2021). CHX (C_22_H_30_C_l2_N_10_) is a broad-spectrum bisbiguanide antiseptic, disinfectant, and preservative that exhibits antimicrobial activity against aerobic and anaerobic bacteria (Kampf 2016; Lin et al. 2021). CHX functions as a bacteriostatic agent at low concentrations and as a bactericidal agent at high concentrations. The primary mode of action involves CHX penetrating the double-layered cell membrane by displacing divalent cations in specific regions, resulting in the formation of gaps between neighboring lipid groups, including LPS (lipopolysaccharides). As a consequence, the cell membrane becomes disrupted (Gregorchuk et al. 2022). In this study, the synergistic activity of CHX and AZM was assessed using *E. coli* strain Crooks (ATCC 8739) as a model. *E. coli* Crooks, an animal pathogen isolated from human feces, has multiple antibiotic resistances (bacdive.dsmz.de/strain/4433) and it is routinely used as a reference strain in testing antimicrobial formulations (Ermawati et al. 2021). To the best of our knowledge, this is the first study aimed to investigate the combinatory effect of CHX with AZM in bacterial growth inhibition. Additionally, we discuss how the combination has the potential to reduce pathogenicity in vitro.

## Materials and methods

### Bacterial strains, culture conditions, and treatments

*E. coli* (ATCC 8739), *E. coli* (DH5α), and *Pseudomonas aeruginosa* (ATCC 27,853) were cultured in Nutrient Broth (NB) or on plates containing 1.5% (w/v) agar in NB at 37 °C. CHX (Chlorhexidine acetate, Merck, Cat no: PHR1222-500MG; Germany) was dissolved in sterile dH_2_O at 1 mg/mL, filter sterilized using polyethersulfone (PES) membrane filter with 0.22 μm pore size, then aliquoted and stored at -20 °C in the dark. AZM (Azithromycin dihydrate; powder for the intravenous solution containing Azithromycin dihydrate 524.1 mg equivalent to 500 mg azithromycin base) was dissolved in sterile dH_2_O at 20 mg/mL. After filter sterilization with a 0.22 μm pore size PES membrane filter, the solution was stored at 4 °C, shielded from light, for a maximum duration of one week, following the guidelines outlined in the prescription.

To determine the minimal inhibitory concentration (MIC), serial dilutions were prepared by adding the antimicrobial agents to the culture media with an OD_600_ of 0.1. Cultures were grown in 15 mL tubes (3 mL volume) in a shaking incubator. OD_600_ values were measured at 8 and 24 h of incubation. For agar spot experiments, after 8 or 24 h of incubation, cultures were serially diluted, and 3 µL of each dilution was spotted on agar plates. The plates were imaged using the G:BOX imaging system with GeneSys image capture software (Syngene; England) (Karaçam and Tunçer 2021).

### Nitroblue tetrazolium (NBT) test

Nitroblue Tetrazolium (NBT) is an artificial electron acceptor and NBT reduction can be used to determine the level of oxidative stress in bacteria (Aiassa et al. 2012; Kalita et al. 2016; Tunçer and Gurbanov 2022). As a result of the oxidative attack, NBT undergoes reduction to form an insoluble blue-black product, formazan which can be detected spectrophotometrically after dissolution (Takeshima et al. 2018).

To assess the effects of the treatments on cellular ROS levels, an NBT assay was conducted as described previously (Tunçer and Gurbanov 2022). Briefly, at the end of the incubation period, 2.5 × 10^7^ bacteria were centrifuged at 13000*xg* for 2 min and then, the supernatants were then removed. The NBT stock solution (Serva, Germany) was prepared at a concentration of 4 mg/mL in dH_2_O and subsequently diluted 1:10 in RPMI-1640 medium (Biological Industries, Israel) and added to the cell pellet as 50 µL. The cells were incubated for 3 h in a shaking incubator at 37 °C with a speed of 160 rpm. After the incubation period, the cells underwent two washes in phosphate-buffered saline (PBS, Biological Industries) and were centrifuged at 16000*xg* for 5 min. The formazan crystals were then dissolved by thorough pipetting in 120 µL of KOH (from a 2 M stock) and 140 µL of DMSO. The absorbances of 200 µL of samples were read colorimetrically at 620 nm in a microplate reader (Multiskan FC, Thermo Scientific, Massachusetts, USA).

### Autoaggregation assays

For Propidium Iodide (PI) staining, samples were centrifuged at 5000 rpm (1844x*g*) for 10 min, and the supernatants were discarded. Bacterial pellets were fixed with 70% ethanol and stained with PI solution (0.1% Triton X-100, 20 µg/mL RNaseA, and 20 µg/mL PI in Phosphate Buffered Saline-PBS) by incubating at room temperature (RT) and a dark environment for 30 min (Tunçer et al. 2018). After incubation, samples were washed and resuspended in 50 µL of PBS. Fluorescence microscopy (Olympus BX53) with a U-FGNA filter (excitation: 535 nm, emission: 617 nm) was used for visualization at 100X magnification, capturing 10–15 images per sample.

The sedimentation assay was applied by following the method by Montero et al. with modifications (Montero et al. 2017). Bacteria were treated with AZM and/or CHX for 24 h. After centrifugation at 5000 rpm (1844x*g*) for 10 min, pellets were resuspended in PBS to an OD_600_ of 1.0. Two tubes were prepared per treatment group, with OD_600_ measurements taken every hour for 8 h. One tube was vortexed, the other remained stationary. For OD_600_ measurement, samples were taken from approximately 1 cm below the surface of cultures.

### Motility analysis

Swarming agar was prepared as defined before (Butler et al. 2010). 15 mL of the swarming agar was poured into 100 mm diameter Petri dishes. After 24 h incubation, bacteria were adjusted to a concentration of 1 × 10^8^ cells/mL based on OD_600_ values. 5 µL of each suspension was pipetted onto the agar plate through the insertion of the pipette tip into the agar. The plates were incubated at 37 °C. The growth was observed and photographed. The colony sizes were measured by ImageJ software (https://imagej.nih.gov).

### Bacterial adhesion assay on HCT-116 epithelial cells in vitro

The in vitro adhesion of *E. coli* to HCT-116 cells was performed as described before with some modifications (Rani et al. 2016). HCT-116 cells were seeded into a 12-well plate in RPMI-1640 medium supplemented with 10% Fetal Bovine Serum (FBS), 2 mM L-glutamine, and 1% penicillin/streptomycin and incubated at 37 °C in a humidified incubator with 5% CO_2_ for 24 h. The following day, when the cells reached approximately 80% confluency, the growth medium was aspirated, and the cells were washed with PBS. *E. coli*, treated with AZM and/or CHX or untreated, were collected, washed, and resuspended in RPMI-1640 medium (without antibiotics and FBS) at a concentration of 1 × 10^8^ cells/mL. For adhesion assay, 500 µL of bacterial suspension was added to the HCT-116 cells and incubated at 37 °C for 3 h. After incubation, the bacterial suspension was removed by pipetting, and the HCT-116 cells were gently washed twice with PBS to remove non-adherent cells. The adhered cells were collected in eppendorf tubes using sterile dH_2_O. Colony Forming Unit (CFU) counting experiments were performed with appropriate dilutions. The bacteria adhered to HCT-116 cells were also visualized by agar spotting assay as described above.

### Protein isolation

To evaluate flagellin (Fli-C) expression in *E.coli* by western blotting, 1 mL of *E.coli* culture, treated with CHX and AZM alone, or in combination, or left untreated, was centrifuged at 5000 rpm (1844*xg*) for 10 min and after the removal of the supernatant, the pellets were washed once with PBS. 200 µL of the lysis buffer (cold PBS containing 0.05% v/v Triton X-100 and 1:100 v/v PMSF-phenylmethylsulfonyl fluoride, prepared as a 100 mM stock) was added to the bacteria pellets and sonication was performed using a sonicator (Bandelin UW 2200; Germany) with 20 cycles of 5 s of sonication at 48% power followed by 5 s of rest while keeping the samples on ice. After centrifugation at 14000x*g* for 15 min at 4 °C, the supernatants (containing the cellular proteins) were transferred to new eppendorf tubes.

Flagellin can activate different cell types that possess TLR5 receptors (Duan et al. 2013) and when flagellin stimulates TLR5, it results in the initiation of NF-κB activation (Olsen et al. 2013; Duan et al. 2013). Thus, HCT-116 cells were analyzed for the expression of p-p65, p65, and TLR5 by western blot after inoculation with *E. coli*. For this, HCT-116 cells were seeded into 6-well plates in a complete RPMI-1640 medium and incubated for 24 h (Rani et al. 2016). Before inoculation with *E. coli*, HCT-116 cells were incubated with an additional 16 h in the complete growth medium but without FBS (Thakur et al. 2016). For inoculation, *E. coli* cells were prepared as described for the adhesion assay above and the epithelial cells were incubated with 1 mL of the bacterial suspension (1 × 10^8^ CFU/mL in RPMI-1640 medium without antibiotics and FBS). After incubation at 37 °C for 3 h, protein isolation from the epithelial cells was performed using T-PER (Thermo Scientific) protein lysis buffer containing protease (cOmplete™ Protease Inhibitor Cocktail, Merck, Germany) and phosphatase inhibitors (cOmplete™, Mini Protease Inhibitor Cocktail, Merck) (Tunçer et al. 2020). Briefly, following the removal of the medium, the cells were washed with cold, cell-culture-grade PBS on ice. A scraper was used to collect the cells to the eppendorf tubes after adding the lysis buffer. Subsequently, the cells in the lysis buffer were incubated on ice for 30 min, with vortexing every 5 min. After the incubation, the proteins were collected by centrifugation at 14000*xg*, 4 °C, for 15 min.

For the quantification of proteins, The Pierce Coomassie Plus Assay Reagent (Thermo Scientific) was employed as per the manufacturer’s guidelines.

### Western blotting

Western blotting was applied as described before (Tunçer and Banerjee 2017). To be noted, for western blotting, proteins obtained from the epithelial cells were loaded into the SDS-PAGE after denaturation at 95 °C, 6 min as described before (Tunçer and Banerjee 2017), while for the flagellin expression, non-denatured *E. coli* proteins were used (Pang et al. 2022).

To analyze Fli-C expression, bacterial proteins were separated in 4% stacking, 10% separating SDS-PAGE gels by running at 100 V, and after running, the proteins were transferred to the Polyvinylidene Difluoride (PVDF) membrane with a pore size of 0.2 μm using Hoefer TE70XP transfer unit (Massachusetts, USA). The semi-dry transfer was applied in the 1X transfer buffer (Transfer Buffer-10X: 250 mM Tris base, 1.92 M glycine. For 1X transfer buffer, mix 100 mL 10X transfer buffer, 200 mL methanol and 700 mL dH_2_O and prechill at 4 °C) for 90 min at 200 mA. As protein loading control for bacterial proteins, the membrane was stained with Ponceau-S solution (0.2 g of Ponceau-S dissolved in 10 mL of acetic acid, volume completed to 200 mL with dH_2_O) and imaged following the transfer (Shao et al. 2020). After removing Ponceau-S completely through washing steps in dH_2_O, the membrane was blocked with 5% v/v non-fat dry milk, prepared in Tris-Buffered Saline containing 0.1% (v/v) Tween20 (TBS-T), followed by the incubation with Fli-C primary antibody overnight at 4 °C. Day after, the membrane was washed 3 times in TBS-T and then incubated with HRP-conjugated secondary antibody at RT for 1 h. At the end of incubation, the secondary antibody was removed and the membrane was washed 3 times in TBS-T. Protein bands were visualized using the Syngene G:BOX imaging system after incubation with ECL (Advansta, San Jose, CA, USA).

For p-p65, p65, and TLR5 expression levels of HCT-116 cells, the same western blot protocol was followed. Glyceraldehyde 3-phosphate dehydrogenase (GAPDH) was used as a protein loading control (Karaçam and Tunçer 2022).

The primary and secondary antibodies used in this study, along with their dilution conditions, are listed in Table S1.

### Statistical analysis

Results are expressed as the mean ± SEM (standard error of the mean). t-test or one-way ANOVA was applied for comparisons (*p ≤ 0.05; **p ≤ 0.01; ***p ≤ 0.001; ****p ≤ 0.0001) using Prism 8.01 (GraphPad, CA, USA). Experiments were repeated independently at least two times with technical replicates.

## Results

The characterization of bacterial pathogenicity involves evaluating the number of infectious bacteria, the ability of the bacteria to adhesion/invasion of host cells, and their impact on the host cells (Duan et al. 2013). Consequently, our investigation focuses on examining the impact of CHZ and AZM treatments, either individually or in combination, on bacterial growth, autoaggregation, flagella expression, and motility. Additionally, we asked how CHZ and AZM treatments influence the bacteria’s ability to adhere to epithelial cells and alter the Toll-Like Receptor 5 (TLR5)-dependent signaling pathway.

### Chlorhexidine and azithromycin act synergistically in growth inhibition and generation of reactive oxygen species

**Fig. 1:**
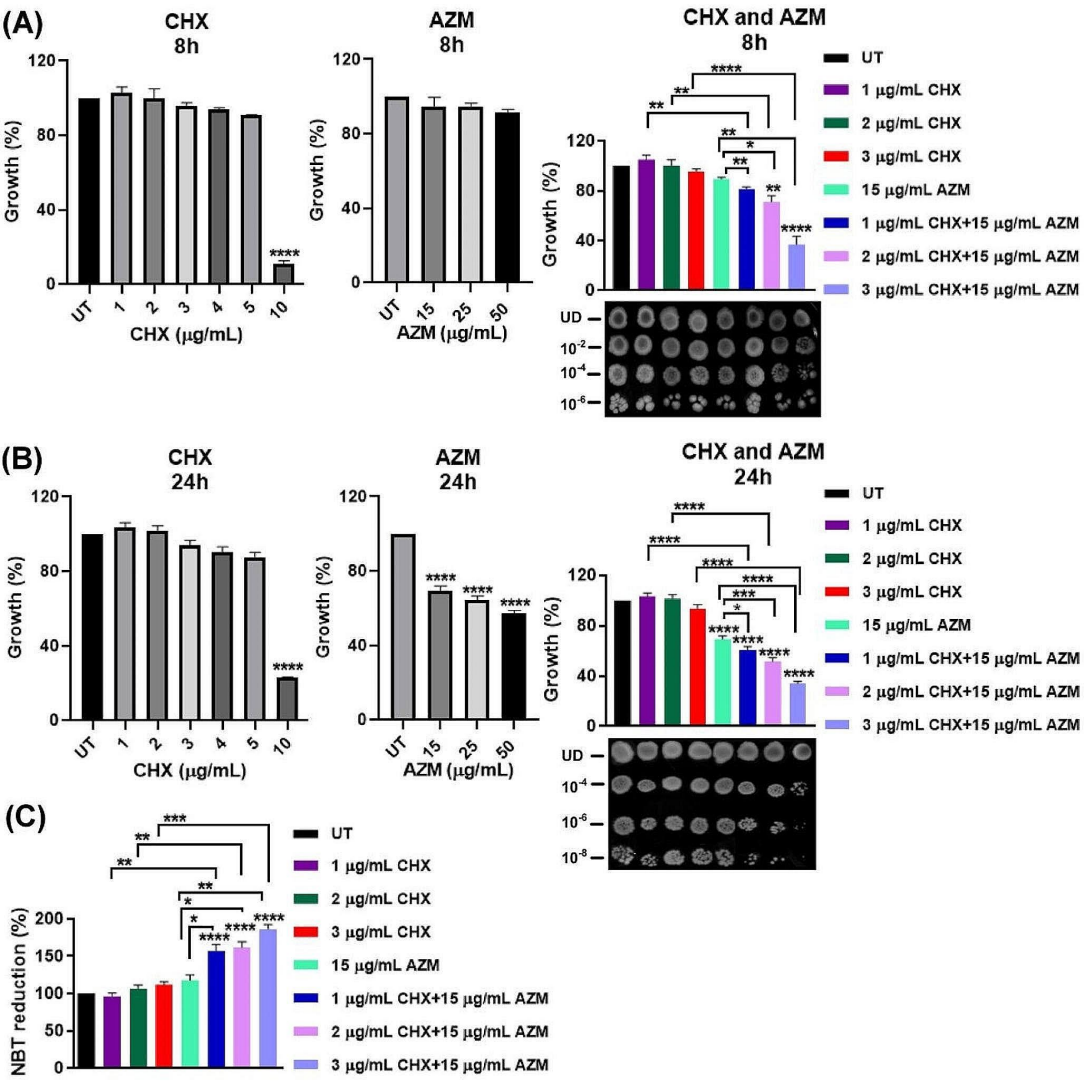
Co-treatment with CHX and AZM enhances growth inhibition. The antibacterial activities of CHX and AZM as single agents or in combination were analyzed on *E.coli* at **A** 8 h and **B** 24 h by measuring optical densities at 600 nm (presented as % respect to the UT) and agar spot plating (lower panels). **C** NBT assay was used to determine the ROS formation after 24 h post-treatment with CHX and/or AZM. The results were given as mean ± SEM. One-way ANOVA was used to compare with UT and t-test was applied for comparisons between the treatment groups as indicated. UT: Untreated; UD: Undiluted

The antibacterial activities of CHX and AZM (alone or in combination) were investigated on *E. coli* strain Crooks (ATCC 8739), which will be referred to as *E. coli* hereafter, using the broth dilution method. The optical densities at 600 nm were measured after 8 h **(**Fig. 1A**)** and 24 h **(**Fig. 1B**)** incubation. Additionally, the antibacterial effects were evaluated by performing agar spot plating represented in the lower panels of Fig. 1A and B. 24 h treatment with 1 or 2 µg/mL CHX did not cause any growth inhibition while the growth of *E.coli* was inhibited only around 8% when treated with 3 µg/mL of CHX. 15 µg/mL AZM alone caused about a 30% reduction in growth. However, when AZM was applied together with 1, 2, or 3 µg/mL of CHX, the inhibition was enhanced by about 39%, 48%, and 66%, respectively. The combined use of CHX and AZM in *E. coli* DH5α strain (Fig. S1) and *P. aeruginosa* (ATCC 27,853) (Fig. S2) has also supported growth inhibition compared to the separate use of these agents. Based on the findings of the growth inhibition experiments, further investigations were carried out using concentrations of 15 µg/mL of AZM and 1, 2, and 3 µg/mL of CHX. Of note, in clinical settings, CHX is used in the concentration range between 0.05 and 4% (Wound Healing and Management Node Group 2017).

Since bacterial cell redox reaction influences the survival of the cells and reactive oxygen species (ROS) can kill pathogens directly by causing oxidative damage to biomolecules (Ong et al. 2017), the oxidative stress in *E. coli* treated with CHX and AZM alone or in combination was analyzed using the NBT assay for a mechanistic insight for the observed anti-bacterial effects. As shown in Fig. 1C, the combined application of CHX and AZM resulted in a drastic increase in ROS formation.

Recent findings from *E. coli* provide evidence suggesting that when the level of secondary ROS damage surpasses a critical threshold, it initiates a self-amplifying process that ultimately leads to the terminal stage of bacterial response to antibiotics (Van Acker and Coenye 2017; Li et al. 2021). Previously, in *A. baumannii* strains, 32 µg/mL CHX treatment (≥ MIC50) resulted in elevated ROS production and enhanced lipid peroxidation. These biochemical changes caused membrane damage and alteration in the membrane proteins, phospholipids, carbohydrates, and nucleic acids (Biswas et al. 2019). In *P. aeruginosa*, incubation with 2 µg/mL AZM (1/64th MIC) was shown to increase the sensitivity of bacteria to H_2_O_2_ treatment by regulating several genes and proteins that are involved in oxidative stress (Nalca et al. 2006). The data presented here suggest that ROS formation can be one of the mechanisms underlying the observed antibacterial effect.

### Chlorhexidine and azithromycin co-incubation results in autoaggregation

**Fig. 2:**
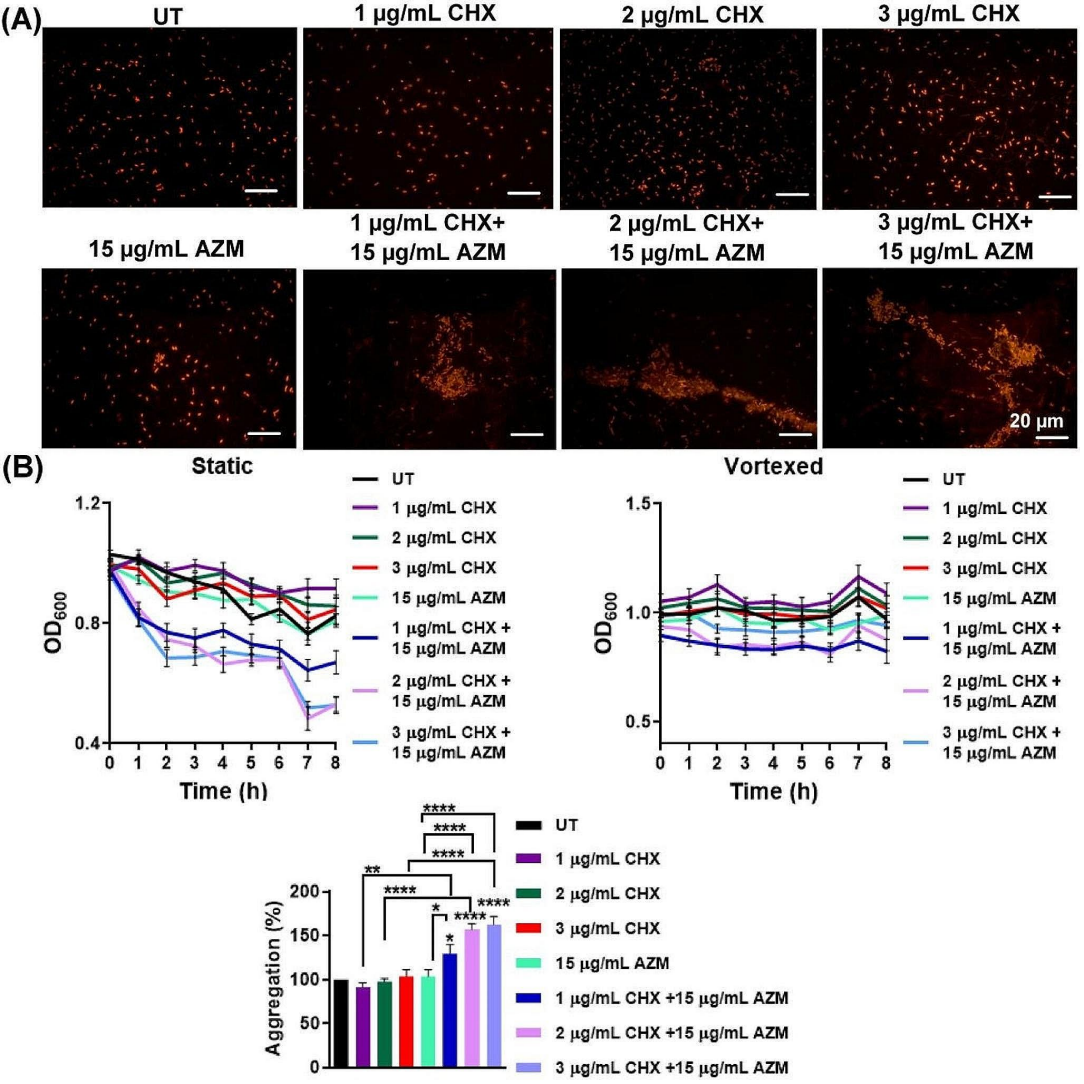
The combined treatment of CHX and AZM enhances autoaggregation. **A** Autoaggregation in *E. coli* was visualized using fluorescence microscopy after PI staining following 24 h CHX and/or AZM treatments. **B** After 24 h incubation with CHX and/or AZM, The OD_600_ values of cultures for each experimental group were provided, where cultures were either left static (on the left) or vortexed (on the right). The percentage of autoaggregation at the 8th h was calculated as a % change with respect to UT (lower panel). Results were represented as mean ± SEM. One-way ANOVA was utilized to compare the groups with the UT group, while t-tests were applied for comparisons among the treatment groups as specified. UT: Untreated

During the incubation of *E. coli* with CHX and AZM, clumps became apparent in the broth culture. Based on this observation, we wanted to determine the effect of CHX and AZM on autoaggregation which refers to the the ability of bacteria to bind to themselves (Trunk et al. 2018). Thus, following 24 h incubation with CHX and AZM alone or in combination, the samples were visualized by fluorescent microscopy after PI staining **(**Fig. 2A**)**. Besides, since the rate of aggregation can be derived from sedimentation, the level of autoaggregation was determined by a sedimentation assay through measuring the optical density of statically incubated or vortexed cultures as described previously (Trunk et al. 2018). The decrease in turbidity in the statically incubated cultures, plotted over time (8 h), was more pronounced in cells co-treated with CHX and AZM, as depicted in Fig. 2B (left panel). As shown in the lower panel of Fig. 2B, the percentage of aggregation after 8 h was significantly higher for *E. coli* cells treated with both CHX and AZM. Bacterial aggregates can contain both live and dead cells as described previously (Monier and Lindow 2003; Schlomann et al. 2019; Petrlova et al. 2020). The discrepancy between the growth inhibition profile in Fig. 1B and the aggregation observed in Fig. 2B (lower panel) implies that the aggregates do not solely consist of dead bacteria. This suggests the possibility that live bacteria may also contribute to the observed aggregation phenomenon.

### Chlorhexidine and azithromycin combination decreases flagellin expression and interferes with swarm motility

The bacterial flagellum is composed of three primary constituents: a basal body, a hook, and an extended filament. This flagellar filament is assembled from numerous repetitions of the protein flagellin (FliC), arranged in a helical configuration, and enclosed at the tip by an oligomeric structure composed of the protein FliD. Flagellins are lengthy proteins that make up the flagellar filament and, except for the FliD protein at the tip, represent the sole structural elements of the filament. *E. coli* genome contains just a single flagellin gene, known as *fliC* (Nedeljković et al. 2021).

**Fig. 3:**
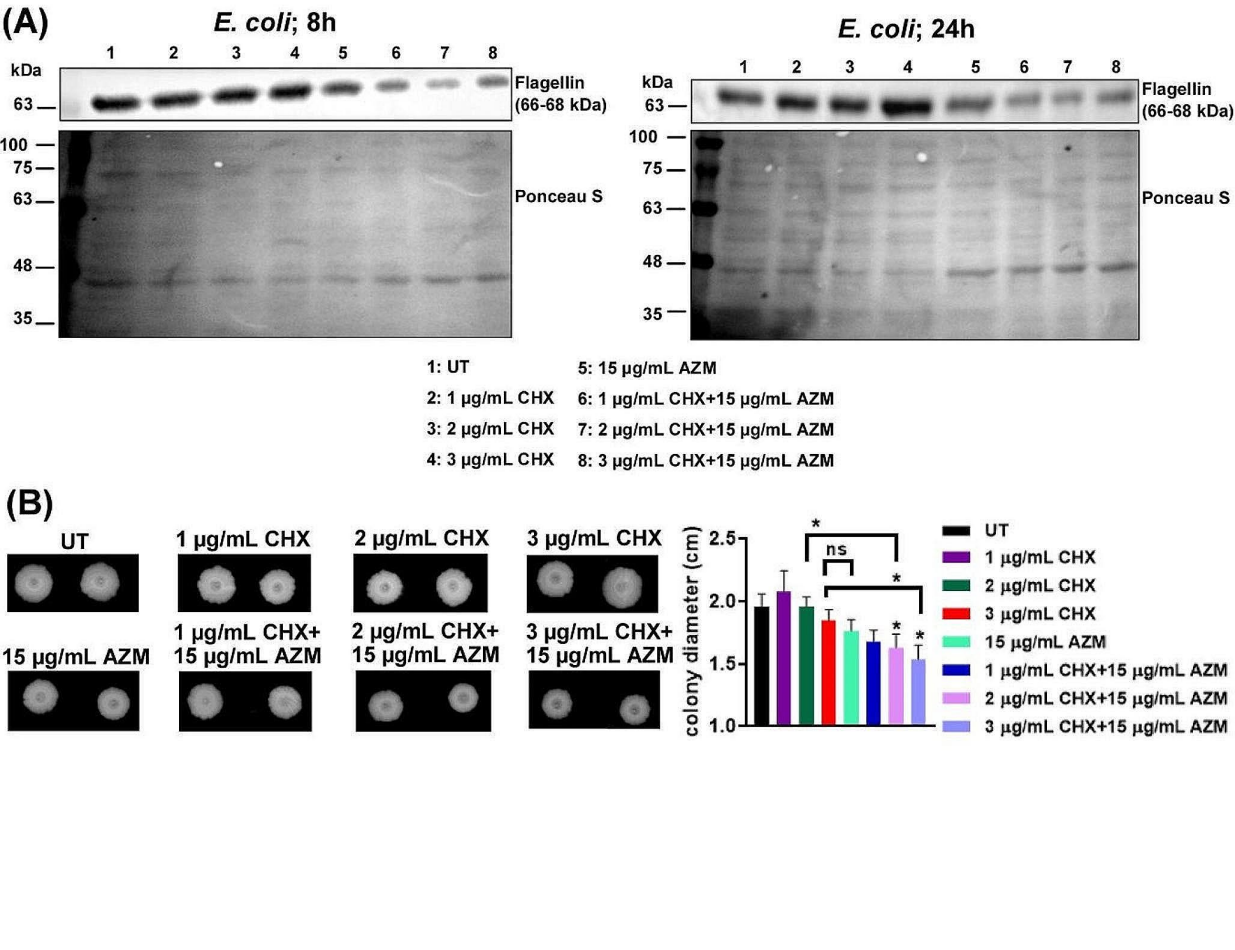
CHX and AZM co-treatment decreases flagellin expression and affects mobility. **A** After 8 h (on the left) and 24 h (on the right) incubation with CHX and AZM (alone or in combination), flagellin expression was analyzed by western blot. Ponceau-S staining was used as the loading control. **B** On the left, the representative colonies for *E. coli* incubated on swarm agar plates for 3 days at 37 °C after treatment with CHX and/or AZM are shown. On the right, the measurements of colony diameters were given. The results were presented as mean ± SEM. t-test was used for the comparison of the colony diameter of untreated (UT) bacteria with the treatment groups. When indicated, the statistics were conducted between the groups

Flagellated bacteria leverage motility to adapt to diverse environmental conditions. Research has revealed that isogenic mutant bacteria lacking flagella exhibit impaired disability in terms of colonization, their ability to cause disease, or both. The virulence of flagellated motile strains and flagellated non-motile strains have been extensively compared in certain bacterial species, convincingly demonstrating the requirement of motility for successful infection (Soutourina and Bertin 2003; Duan et al. 2013). In this respect, we investigated how the expression of flagellin, the basic subunit that polymerizes to form the rigid flagellar filament of *E. coli* (Nedeljković et al. 2021), is modulated upon treatment with CHX and AZM. Figure 3A shows that flagellin (FliC) expression decreased in the bacteria co-treated with CHX and AZM. Exposure to subinhibitory concentrations of macrolides, including AZM has been previously shown to reduce the flagellin expression and motility in *P. aeruginosa* and *Proteus mirabilis* (Molinari et al. 1992; Kawamura-Sato et al. 2000; Nalca et al. 2006). Here we show that although AZM inhibits the flagellin expression when it was applied together with CHX, the decrease in the expression was much more prominent.

The virulence of various important human pathogens has been associated with swarm motility, which contributes to antibiotic resistance (Allison et al. 1994; Gardel and Mekalanos 1996; Macfarlane et al. 2001; Overhage et al. 2008; Lai et al. 2009; Birhanu et al. 2018). Swarming motility refers to the bacterial movement across a solid surface, facilitated by the rotational motion of flagella (Patrick and Kearns 2012). Based on the results indicating the decreased flagellin expression in AZM-treated and CHX and AZM co-treated bacteria, we sought to determine the effect of CHX and AZM, both individually and in combination, on the motility of *E. coli* (Fig. 3B). After 72 h incubation, colony morphologies were visualized and photographed (on the left), and the swarm colony diameter was measured (right panel). It can be seen that albeit not drastic, there was a reduction in colony diameters when CHX and AZM were applied together. It can be inferred that the observed decrease in motility (Fig. 3B) is, to some extent, a consequence of the reduced flagellin expression. It should also be emphasized that, despite a significant reduction in flagellin expression (Fig. 3A), the bacteria still retained their swarming ability. Since flagellin expression was not abolished completely, the result may be attributed to the remaining flagellin activity and also presence of functional flagellar components. Nalca et al. described that sublethal AZM concentration down-regulated the expression of various proteins required for flagellum biosynthesis, in addition to flagellin, and also reduced flagellum-driven motility (Nalca et al. 2006). Accordingly, our results also show an AZM-dependent decrease in flagellin expression which was further down-regulated in the presence of CHX. Further research is needed to investigate how the treatment with AZM and CHX alone or in combination alters the expression of the other flagellar proteins and affects motility.

Many environmental and pathogenic bacteria prefer to exist as multicellular structures and exhibit the property of autoaggregation under conditions of environmental stress, such as toxins, antibiotics, predation, or lack of nutrients (Trunk et al. 2018). Ulett et al. showed that when flagella are present in low numbers, autoaggregation, mediated by Ag43 (a surface-displayed autoaggregation protein), decreases the motility of *E. coli*. On the other hand, the authors claimed that increased flagellation may create a physical barrier that prevents the intimate contact required for Ag43-Ag43 interaction; a balance exists between flagellation and autoaggregation, and flagella-driven bacterial movement may reduce the efficiency of Ag43-mediated aggregation (Ulett et al. 2006). This phenomenon was observed in *E. coli* as phase variants expressing and not expressing Ag43 revealed contrasting motility phenotypes (Ulett et al. 2006). It should be emphasized that we observed a drastic increase in autoaggregation when CHX and AZM were used concurrently (Fig. 2), but not a profound loss in motility when CHX and AZM were applied alone or in combination. Therefore, one can suggest that Ag43-dependent aggregation occurs in subpopulations of *E. coli* and affects the bacterial motility in these subpopulations (Ulett et al. 2006). Supporting our results, Coquet et al. demonstrated that in *E. coli*, incubation with CHX resulted in a significant up-regulation in the Ag43 protein level (Coquet et al. 2017).

Here, it is also worth noting that the formation of aggregates into microcolonies or biofilms can favor adherence, but at the same time impedes the elimination of the microbial population since the aggregated cells can be more efficiently phagocytosed by the host (Galdiero et al. 1988). As described previously, the *E. coli* strain ATCC 8739 (Crooks) is a commonly used laboratory reference strain for growth inhibition experiments (Nassima et al. 2019), however, it is a poor biofilm-forming bacteria (Król et al. 2019). Future studies involving a biofilm model bacterium can shed light on the effects of the combination on aggregation, biofilm formation, as well as the host’s interference with the aggregates.

### Adherence to the epithelial cells diminishes with the co-treatment

**Fig. 4:**
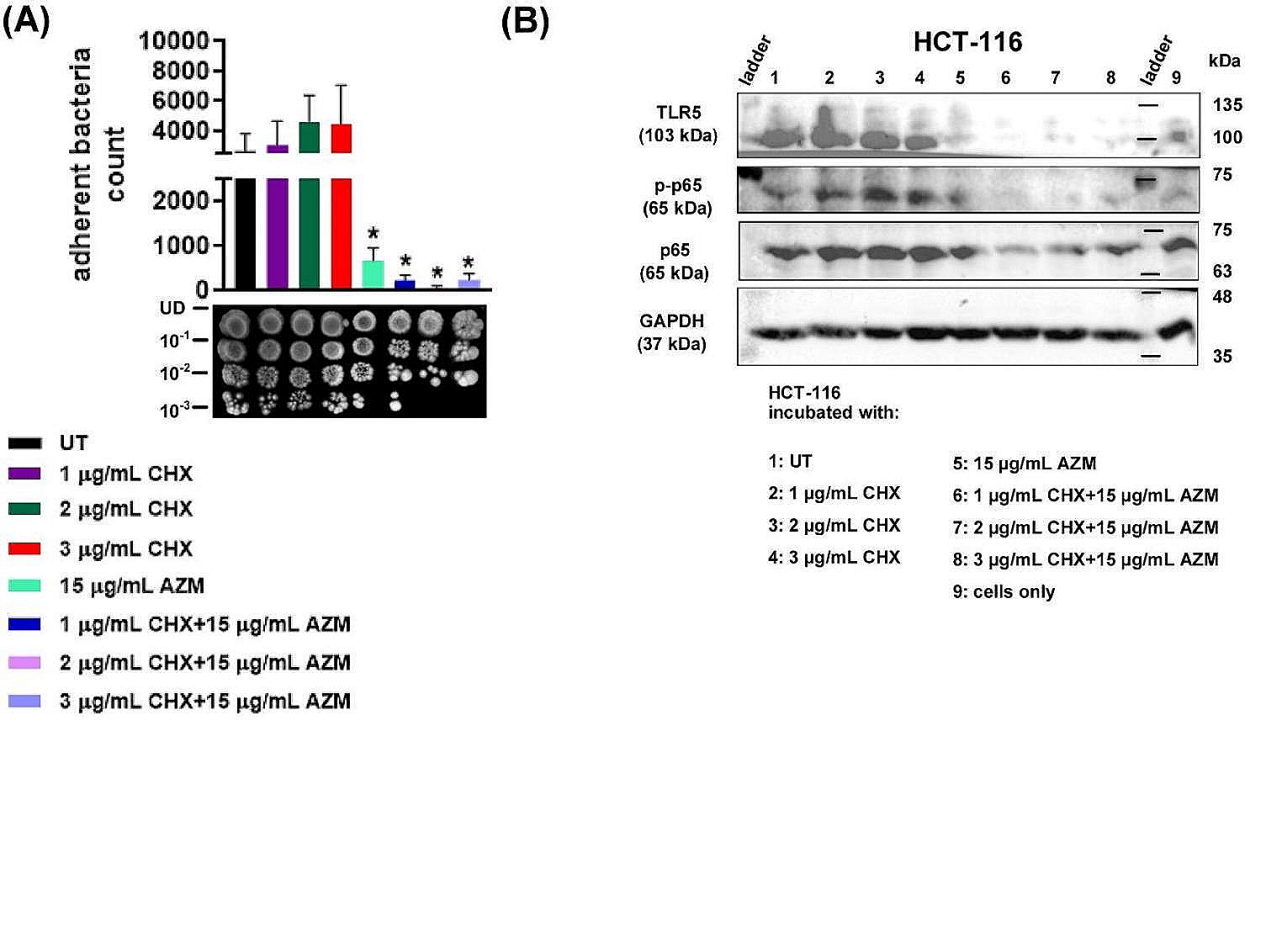
The combined application of CHX and AZM reduces the adhesion of *E. coli* to epithelial cells. After treatment with CHX and/or AZM, *E. coli* were co-cultured with HCT-116 cells for 3 h. **A** The number of bacteria adherent to the epithelial cells was counted (upper panel) and also spotted on the agar plate (lower panel). t-test was applied for comparison with untreated (UT) bacteria. UD: Undiluted. **B** TLR5 expression and p65 expression and activation in the epithelial cells incubated with bacteria were shown. Cells cultured under the same conditions without bacterial incubation were used as the control group (cells only). GAPDH was used for loading control

Flagellum is also important for increasing the pathogen-host interactions and promoting subsequent adherence and colonization, and this feature contributes to the main role of flagella in pathogenesis (Duan et al. 2013; Kalita et al. 2014). Several instances have been observed in which flagellin serves as the adhesive component in various *E. coli* strains (Chaban et al. 2015). Thus, we asked if decreased flagellin expression in CHX and AZM co-treated cells impairs the ability of bacteria to adhere to epithelial cells. For this, CHX and/or AZM-treated bacteria were incubated with the colorectal cancer epithelial cells HCT-116 as a model (Thakur et al. 2016). After co-culturing, the number of bacteria adherent to epithelial cells was determined. As shown in Fig. 4A, CHX and AZM co-treatment reduced the adherence capacity of the *E. coli* which was further confirmed by spot plate assay (lower panel).

Flagellin can stimulate a variety of TLR5-expressing cell types (Duan et al. 2013). The expression level of TLR5 is directly correlated with bacterial adhesion to the epithelial cells (Kalita et al. 2014). Stimulation of TLR5 by flagellin leads to the activation of NF-κB (Olsen et al. 2013; Duan et al. 2013). As demonstrated in Fig. 4B, the activation of p65 was reduced in HCT-116 cells when they were incubated with bacteria that were co-treated with CHX and AZM. In murine osteoblasts, expression of TLR5 was shown to be upregulated following exposure to the TLR5 agonist flagellin (Madrazo et al. 2003). Similarly, here we show TLR5 expression is also induced in the epithelial cells incubated with *E. coli* compared to the cells not incubated with the bacteria. Nonetheless, the upregulation of TLR5 expression was found to be reversed in cells incubated with the bacteria treated with AZM alone or CHX and AZM combination, which aligns with the observed decline in flagellin expression in bacteria treated with AZM or co-treated with CHX and AZM (Fig. 3A). It is noteworthy that inhibition of TLR5 expression was more prominent in the cells incubated with bacteria that were co-treated with CHX and AZM.

## Discussion

Based on the results obtained from this study, we put forth the proposition that sub-lethal concentrations of CHX can enhance the cellular accumulation of AZM. It is known that when CHX is present in low concentrations, it can cause membrane perturbation and increase membrane permeability by attaching to LPS and membrane phospholipids. This property of CHX may facilitate the uptake of AZM. Subsequently, dissipation of the proton motive force and impairment of the respiration chain may enhance the intracellular ROS production (Xia et al. 2021) and also favor the transcriptional and translational effects of the antibiotic (Nalca et al. 2006; Konikkat et al. 2021) by increasing its intracellular accumulation. AZM has an approximate diameter of 1.16 nm (1.3 × 1.0 × 0.92 nm cuboid structure) and it is stated that it does not pass effectively through porin channels. However, it enters the cell with the support of its structure, known as “self-promoted uptake” (Farmer et al. 1992; Myers and Clark 2021). For CHX, which disrupts the outer membrane integrity in Gram-negative bacteria at sub-lethal concentrations, the biguanide groups strongly interact with exposed anionic regions on the cell membrane and cell wall, particularly acidic phospholipids and proteins, and this binding leads to the displacement of divalent cations (Mg^2+^, Ca^2+^). However, the rigidity of CHX’s 6-carbon hydrophobic region prevents it from folding adequately to penetrate the cell bilayer. Therefore, CHX forms bridges between adjacent phospholipid headgroup pairs, each of which is attached to a CHX molecule’s biguanide, effectively replacing the relevant divalent cations. At higher concentrations of CHX on the other hand, the interactions are more pronounced leading to the transition of the membrane into a liquid crystalline state, loss of structural integrity, and catastrophic leakage of cellular materials. It is also noted that this mechanism is used to explain why acquired resistance to CHX is less common and does not emerge rapidly: the effect of multidrug efflux pumps can soften the efficacy of quaternary ammonium compound-based biocides at low concentrations, but the insolubility of bisbiguanides in the membrane’s hydrophobic core reduces the impact of efflux pumps on CHX efficacy (Gilbert and Moore 2005). AZM, being an amphiphilic molecule capable of interacting with both the hydrophobic and hydrophilic regions of the lipid monolayer (Montenez et al. 1996; Berquand et al. 2004), can utilize the hydrophilic regions facilitated by CHX to aid its own transmembrane transport. At sub-inhibitory concentrations, cationic CHX molecules can interact with negatively charged LPS regions, effectively “locking” into them. Therefore, the outer membrane of Gram-negative bacteria acts as a permeability barrier for CHX, limiting its antibacterial activity as it cannot reach the cytoplasmic membrane (Cieplik et al. 2019). Similarly, it is proposed that the electrostatic interaction between AZM and the negatively charged heptose-phosphate region of LPS in the outer membrane structure of Gram-negative bacteria also forms a permeability barrier, preventing effective entry of AZM into the cell (Vaara 1993). However, the simultaneous utilization of these two agents in Gram-negative bacteria may transform the drawback of each agent into a benefit. This can be attributed to the possible creation of a hydrophilic interaction zone, which could enhance the entry of AZM into the cell, thereby potentially increasing its effectiveness. Moreover, CHX treatment can alter membrane properties, potentially affecting the characteristics (such as folding, organization, functionality, etc.) of membrane-spanning outer membrane porins (OMPs). To illustrate, the structure and mechanical properties of LPS are suggested to be important for OMP folding. Additionally, the processes involving how the membrane is inserted and how lipids are arranged affect how fast OMPs fold (Horne et al. 2020). It is important to note that macrolides can also traverse the outer membrane barrier by utilizing channels formed by porins as shown by the work of Capobianco and Goldman (Capobianco and Goldman 1994). This means that the changes brought about by CHX in the bacterial outer membrane could help AZM get inside the bacteria more effectively by adjusting how these channels work. To truly understand how CHX and AZM work together, we need thorough research to uncover the detailed mechanisms behind this partnership.

Regarding the impact on the host cells, TLR5 expression decreased in the epithelial cells incubated with CHX and AZM co-treated bacteria compared to the untreated bacteria. In those cells, expression and activation of p65 were also diminished. The underlying cause for this effect can be ascribed to the decreased expression of flagellin, which acts as a potent ligand for TLR5. Furthermore, the decreased expression of flagellin in bacteria and TLR5 in epithelial cells may both contribute to reduced bacterial adherence to the epithelial cells.

## Conclusion

These findings propose the potential for bacteria treated simultaneously with AZM and CHX to manifest a less virulent phenotype (Yang and Yan 2017). The pairing of CHX and AZM presents a potential strategy for curbing the incidence of hospital-acquired infections, such as those connected with catheters (Lin et al. 2021). Additionally, when coupled with the topical applications of AZM (McHugh et al. 2004; Hajheydari et al. 2011; Abtahi-Naeini et al. 2021), CHX shows promise for the treatment of skin infections, and infected wounds, in addition to oral care. To obtain more detailed information, performing in vivo studies is needed. Besides, a comprehensive investigation is required especially to examine the effects of aggregation as a bacterial survival mechanism, and precise immunogenic alterations of the treatment, as well as its impact on epithelial cells and/or phagocytic cells, warrants detailed investigation.

### Electronic supplementary material

Below is the link to the electronic supplementary material.

Supplementary Material 1

## Data Availability

All data generated during this study are available from the corresponding author (sinem.tuncer@bilecik.edu.tr) upon reasonable request.

## Abbreviations

AIDS: Acquired Immunodeficiency Syndrome
AMR: Antimicrobial Resistance
AZM: Azithromycin
CFU: Colony Forming Unit
CHX: Chlorhexidine
FBS: Fetal Bovine Serum
OMPs: Outer Membrane Porins
PBS: Phosphate Buffered Saline
PI: Propidium Iodide
ROS: Reactive Oxygen Species
RT: Room Temperature
WHO: World Health Organization
