# Supplementary material for: Unveiling the synergistic potency of chlorhexidine and azithromycin in combined action
Source: Naunyn Schmiedebergs Arch Pharmacol. 2024 Feb 20;397(8):5975–87. doi: 10.1007/s00210-024-03010-0 (PMC11329591; doi:10.1007/s00210-024-03010-0)
Supplement: Supplementary file 1 — Supplementary Material 1 [file 210_2024_3010_MOESM1_ESM.docx]

**Supplementary Information**

**Unveiling the Synergistic Potency of Chlorhexidine and Azithromycin in Combined Action**

Gizem Samgane^1^, Sevinç Karaçam^1,2^, Sinem Tunçer Çağlayan^3^

^1^ Department of Biotechnology, Bilecik Şeyh Edebali University, 11100 Bilecik, Turkey

^2^ Central Research and Application Laboratory, Bilecik Şeyh Edebali University, 11100 Bilecik, Turkey

^3^ Department of Medical Services and Techniques, Vocational School of Health Services, Bilecik Şeyh Edebali University, 11100 Bilecik, Turkey

**Correspondence**

S. Tunçer Çağlayan, Department of Medical Services and Techniques, Vocational School of Health Services, Bilecik Şeyh Edebali University, Pelitözü Mah. Fatih Sultan Mehmet Bulvarı No:27, 11100 Bilecik, Turkey

Tel: +90-228-214-1766

E-mail: [sinem.tuncer@bilecik.edu.tr](mailto:sinem.tuncer@bilecik.edu.tr)

ORCID: 0000-0002-8947-8646

| **Primary Antibody** | **Dilution** | **Vendor and Catalog No** |
| --- | --- | --- |
| FliC | 1:3000 in skim milk | Biorbyt, Cat no: orb240580 |
| TLR5 | 1:300 in BSA | Santa Cruz Biotechnology, Cat no: 517439 |
| p65 | 1:300 in BSA | Santa Cruz Biotechnology, Cat no: sc-8008 |
| p-p65 | 1:300 in BSA | Santa Cruz Biotechnology, Cat no: sc-136548 |
| GAPDH | 1:1000 in skim milk | Santa Cruz Biotechnology, Cat no: sc-25778 |
| **Secondary Antibody** | **Dilution** | **Vendor and Catalog No** |
| Goat Anti-Rabbit IgG | 1:1000 in skim milk | Bioss, Cat no: bs-0295G-HRP |
| Donkey Anti-Mouse IgG | 1:5000 in skim milk | Santa Cruz Biotechnology, Cat no: sc-2314 |

**Table S1.** Primary and secondary antibodies used for western blotting


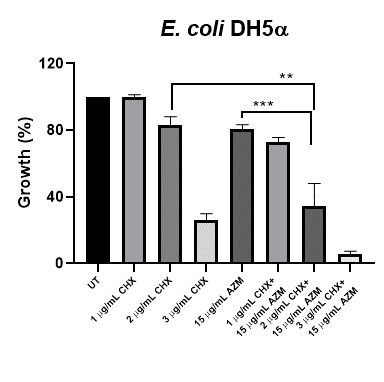


**Fig. S1.** Co-treatment with CHX and AZM enhance growth inhibition. The antibacterial activities of CHX and/or AZM were analyzed on *E.coli* DH5α after 24 h incubation by measuring optical densities at 600 nm. The results were presented as % respect to the UT. t-test was used for the comparisons.


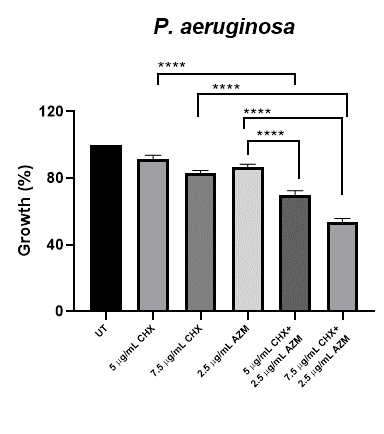


**Fig. S2. The combined treatment of CHX and AZM enhances** growth inhibition. The antibacterial activities of CHX and/or AZM were assessed on *P. aeruginosa* after 24 h of incubation by measuring optical densities at 600 nm. The results were expressed as percentages relative to the untreated (UT) group. t-tests were employed for the comparisons.
